# Systems Pharmacology-Based Precision Therapy and Drug Combination Discovery for Breast Cancer

**DOI:** 10.3390/cancers13143586

**Published:** 2021-07-17

**Authors:** Ze-Jia Cui, Min Gao, Yuan Quan, Bo-Min Lv, Xin-Yu Tong, Teng-Fei Dai, Xiong-Hui Zhou, Hong-Yu Zhang

**Affiliations:** 1 Hubei Key Laboratory of Agricultural Bioinformatics, College of Informatics, Huazhong Agricultural University, Wuhan 430070, China; zejia_cui@pku.edu.cn (Z.-J.C.); gm@webmail.hzau.edu.cn (M.G.); quanyuan@mail.hzau.edu.cn (Y.Q.); lbm612@webmail.hzau.edu.cn (B.-M.L.); tongxinyu@webmail.hzau.edu.cn (X.-Y.T.); 2 Lab of Epigenetics and Advanced Health Technology, Space Science and Technology Institute (Shenzhen), Shenzhen 518117, China; 3 AtaGenix Laboratory, Wuhan 430070, China; tfdai@atagenix.com

**Keywords:** precision drug discovery, breast cancer, breast cancer subtypes, drug combination, systems pharmacology

## Abstract

**Simple Summary:**

Breast cancer (BC) is a typical global cancer and the second leading cause of cancer-related deaths among women worldwide. BC is a heterogeneous disease with several subtypes, and it is a challenge to use multi-omic data effectively to find suitable drugs for patients. In this paper, we used the GeneRank algorithm and gene dependency network to propose a precision drug discovery strategy that can recommend drugs for individuals and screen existing drug combinations that could be used to treat different BC subtypes. Our results showed that this precision drug discovery strategy identified important disease-related genes in individuals and specific groups, supporting its efficiency, high reliability, and practical application value in drug discovery.

**Abstract:**

Breast cancer (BC) is a common disease and one of the main causes of death in females worldwide. In the omics era, researchers have used various high-throughput sequencing technologies to accumulate massive amounts of biomedical data and reveal an increasing number of disease-related mutations/genes. It is a major challenge to use these data effectively to find drugs that may protect human health. In this study, we combined the GeneRank algorithm and gene dependency network to propose a precision drug discovery strategy that can recommend drugs for individuals and screen existing drugs that could be used to treat different BC subtypes. We used this strategy to screen four BC subtype-specific drug combinations and verified the potential activity of combining gefitinib and irinotecan in triple-negative breast cancer (TNBC) through in vivo and in vitro experiments. The results of cell and animal experiments demonstrated that the combination of gefitinib and irinotecan can significantly inhibit the growth of TNBC tumour cells. The results also demonstrated that this systems pharmacology-based precision drug discovery strategy effectively identified important disease-related genes in individuals and special groups, which supports its efficiency, high reliability, and practical application value in drug discovery.

## 1. Introduction

Breast cancer (BC) is a solid tumour that seriously endangers women’s health and is the most common type of cancer. Globally, there are approximately 1 million to 1.3 million newly diagnosed cases each year [1,2,3]. BC is a heterogeneous disease with subtypes that differ in morphology, molecular biology, clinical manifestations, and response to treatment [4]. Due to the high heterogeneity of BC patients [5], the genes that are related to drug response may not be the same among patients, even among those with the same subtype. In addition, genomic and environmental changes, as well as other factors, should be considered for each patient in precision medicine [6]. Therefore, it is difficult to identify a single genetic variation or gene signature to predict the drug response of all patients with BC.

Precision medicine, which aims to tailor individualized treatment for each patient based on the person’s genes, lifestyle, and environment, represents a promising direction in medicine [7,8]. In cancer, this strategy promises to identify drugs that could target patients’ genetic variations [9]. Therefore, it is essential to predict the effects of drugs in specific cancer patients or in patients with specific subtypes of cancer.

Most current personalized therapy strategies depend on biomedical big data and statistically derived information on genetic variations [10]. These methods generally use a genome-wide association study (GWAS) or other tools to identify the genetic variations related to drug response. The effect of the drug on the patient is judged by whether the patient has the corresponding genetic variation [11]. However, this paradigm faces some fundamental difficulties in practice. First, complex diseases such as cancer may be caused by genetic variations at many sites, not by a simple single nucleotide polymorphism (SNP) or other genetic variation [12,13]. Therefore, it is difficult to identify driver genes. Second, even though some true genetic variation sites have been identified, the penetrance of the variants is usually unknown, which hinders therapeutic decision-making [14,15]. There are some methods that aim to identify gene signatures whose expression levels are correlated with treatment response and survival following therapy with specific drugs [16,17]. However, prediction models established with statistical or machine-learning methods are specific to the data sets used to design them and thus have poor generalization capability [18]. One prediction model is also needed for each drug using these methods, which is inefficient.

The essence of medical therapy is to use drugs to target disease-driven genes. Drugs with the same indication may have hugely different target spectra. Patients exhibiting the same disease symptoms may have distinct underlying risk factors, which can be reflected by their gene expression profiles. Therefore, it may be promising to identify the key genes for each patient based on the gene expression data of the patient. Then, the effect of a drug on a specific patient could be inferred by testing whether the drug could target the important genes of the corresponding patient [19].

Recently, network biology, which can reveal the gene regulatory relations among complex systems, has been successfully applied to identify important genes for complex diseases [20,21]. Our previous work proposed a new method for constructing a gene dependency network using gene expression and clinical data (survival time and survival status) of cancer patients [22]. This network could reveal gene dependencies during the process of phenotypic changes and was proven to facilitate the selection of prognostic genes in cancer. GeneRank [23,24], which can capture both the network topology of the biological network and the initial importance of the nodes in the network, was proposed to prioritize important genes in biological systems. In this work, we propose a systems pharmacology-based drug discovery strategy. We applied our network-inferring algorithm, which is based on gene expression data and the corresponding prognostic information of cancer patients, to reconstruct the gene dependency network, which revealed the gene dependencies related to cancer prognosis. Subsequently, a modified GeneRank algorithm, which could be used in a direct network, was applied to identify the important genes in the gene dependency network. For each patient, the initial importance of the genes was evaluated according to the fold-change between the gene expression levels of the cancer tissue and those of the control tissue (e.g., adjacent normal tissue). As the gene dependency network can reveal gene dependencies in the process of cancer progression, and the fold-change can represent the differentially expressed genes in patients, our method may identify the important genes in cancer prognosis for each patient. After determining the important genes for each patient, the effect of each drug in the patient was predicted with the Kolmogorov–Smirnov test. The effect of a drug was judged by testing whether the important gene list of the patient was enriched for the targets of the drug.

Multiple factors affect the occurrence of complex diseases [25,26,27]. For example, changes in multiple genes and pathways cause the occurrence of cancer. Therefore, treatment with a single drug may not produce a significant effect. In addition, the stimulation of a single target by a drug can induce excessive activation or inhibition of a biological target in the human body that will also affect other normal human functions and even activate anti-protection mechanisms or bypass compensation mechanisms, resulting in toxic side effects and drug resistance [28]. Compared with single drug treatment, drug combinations improve the diagnosis and treatment effect while reducing side effects [29,30].

We aimed to identify valuable drug combinations for BC treatment and to verify the effectiveness of the precision drug discovery strategy proposed in this study. We predicted four drug combinations for patients with specific subtypes of BC and validated the effects of the drug combinations in vivo and in vitro.

## 2. Materials and Methods

### 2.1. Data Sources

The data sets of BC [31], ovarian cancer (OV) [32], and glioblastoma multiforme (GBM) [33] were downloaded from TCGA (https://portal.gdc.cancer.gov/, accessed on 12 August 2017). Each data set contained the mRNA expression profiles, drug treatment data, and prognosis information for a large number of cancer patients (https://portal.gdc.cancer.gov/projects, accessed on 12 August 2017). In addition, the mRNA expression data for the control samples of each cancer were collected from TCGA. The mRNA expression data of OV and GBM samples were obtained with Agilent gene chips, and level 3 data were used in this paper. For BC, the number of segments per kilobase of exon per million mapped reads (FRKM) for each gene from RNA sequencing (RNA-seq) data was used. The details for these data are shown in Table S1. When we constructed the gene dependency network for each cancer, the gene expression data as well as the prognosis information of each patient were binarized. For each gene, the expression level in a sample was set as 1 or 0, depending on whether it was higher or lower than the median gene expression level across all samples. The prognostic risk for each patient was also set as high-risk (denoted by ‘1’) or low-risk (denoted by ‘0’). If a patient died within a certain time frame (set as 1825 days for BC, 1200 days for OV, and 400 days for GBM to ensure that the number of patients in the two groups for each cancer was similar), the patient was designated as high-risk; if a patient survived for longer than the indicated time period, the patient was designated low-risk; if survival information was not available the patient was excluded from this analysis (network construction).

Known cancer genes were collected from the Human Gene Database (GeneCards, http://www.genecards.org/, accessed on 23 October 2017) [34]. In this work, for BC, OV, and GBM, corresponding cancer genes with scores of no less than 20 were selected as known cancer genes. The targets for each drug were extracted from the Drug–Gene Interaction database (DGIdb, http://dgidb.genome.wustl.edu/, accessed on 27 March 2017) [35], the Therapeutic Target Database (TTD, http://bidd.nus.edu.sg/group/cjttd/, accessed on 27 March 2017) [36], and DrugBank (https://www.drugbank.ca, accessed on 27 March 2017) [37]. For each drug combination, the combined set of the targets collected from all three data sets was used as the target list. All the candidates for drug combinations and obsolete agents for cancer were obtained from the Drug Combination Database (DCDB, http://www.cls.zju.edu.cn/dcdb/, accessed on 18 May 2018) [38] and ClinicalTrials.gov (https://clinicaltrials.gov, accessed on 18 May 2018).

### 2.2. Construction of Gene Dependency Network

In our previous work, we proposed a method [22] to construct a gene dependency network which could reveal the gene dependencies related to cancer prognosis. Here, we constructed gene dependency networks for BC, OV, and GBM. The gene dependency network for each kind of cancer was constructed as follows:

1. For each candidate gene pair (denoted as ‘A’ and ‘B’) and the prognostic information (survival time and survival status), the gene expression levels and the prognosis information of each patient were binarized.
2. The gene dependency value of gene A to gene B in the context of prognosis (P) was calculated with conditional mutual information (CMI (A, P|B)).
3. A total of 100,000 gene pairs were selected randomly and 1,000,000 gene dependency values were calculated as the null hypothesis distribution.
4. Based on the null hypothesis distribution, the z-test was applied to calculate the significance of the gene dependency value of gene A to gene B in the context of prognosis (P). In this work, gene pairs with *p*-values less than 1 × 10^−5^ were considered significant pairs.
5. All significant gene pairs were used to construct the gene dependency network.

### 2.3. Modified Algorithm of GeneRank

The GeneRank algorithm [24], derived from PageRank [23], which concerns both the topological structure of the biological network and the importance of the nodes in the network, can successfully prioritize the genes in a network. GeneRank was previously applied to define an indirect network. Here, we adopted and extended the strategy of our previous work [39] and applied GeneRank to define a direct network. The modified algorithm of GeneRank can be described as follows:

(1) $$r_{j}^{n}=\left(l-d\right)f_{j}+d\overset{N}{\underset{i=1}{\sum }}\frac{w_{ij}r_{i}^{n-1}}{deg_{i}}$$

where $r_{j}^{n}$ and $r_{i}^{n-1}$ were the score of gene *j* after *n* iterations and the score of gene *i* after *n−1* iterations, respectively; $f_{j}$ was the initial score of gene *j*, which was set as the absolute value of the fold-change of the gene between the disease sample and the controls; *w* was the adjacent matrix of the gene dependency network, $w_{ij}$ = 1 if gene *i* was significantly dependent on gene *j*, otherwise,$w_{ij}$ = 0; *deg_i_* described how many nodes were dependent on gene *i*, that is, the out-degree of gene *i*; *N* was the number of genes in the network; and *d* (0 ≤ *d* < 1) weighted the gene dependency network. In this work, we set *d* as 0.30 (when this algorithm was applied in BC, *d* was set as 0.85, which is the same as the original method). The iteration of the algorithm stops until ε < 0.00001, while ε was one-norm of $\left|r_{j}^{n}\;-\;r_{j}^{n-1}\right|$.

### 2.4. Drug Combination Prediction for Patients with Specific BC Subtypes

Four drug combinations were identified using this strategy for drug discovery for patients with specific subtypes of BC. Three of the drug combinations, i.e., irinotecan and sunitinib, cisplatin and topotecan, and gefitinib and irinotecan, were derived from the DCDB. The combination of exemestane and idarubicin was derived from ClinicalTrials.gov (https://clinicaltrials.gov/, 18 May 2018). In preclinical colorectal cancer models, the effect of the combination of irinotecan and sunitinib was superior to that of either single agent alone [40]. A randomized phase III trial demonstrated a survival advantage in cervical carcinoma patients treated with combination chemotherapy with cisplatin and topotecan [41]. Gefitinib plus irinotecan can be used to treat advanced fluoropyrimidine-refractory colorectal cancer [42]. Based on the indication of ClinicalTrials.gov (https://clinicaltrials.gov/, accessed on 18 May 2018), exemestane combined with idarubicin may have synergistic activity in the treatment of urothelial carcinoma, bladder cancer, or urinary bladder neoplasms.

### 2.5. Enrichment Analysis

To validate whether the important genes in our gene dependency network are cancer related, we applied gene set enrichment analysis (GSEA) [43] to test whether the genes common to all the data sets and the enriched Kyoto Encyclopedia of Genes and Genomes (KEGG) pathways were significant in cancer.

The Kolmogorov–Smirnov test was applied to test whether the drug targets (known cancer genes) were enriched among the top-ranked genes by our method.

### 2.6. Cells and Reagents

The human BC cell lines MCF-7, SK-BR-3, and MDA-MB-231 were purchased from the China Center for Type Culture Collection (Wuhan, Hubei, China), and all three cell lines were checked for mycoplasma. MCF-7 and MDA-MB-231 cells were cultured in high-glucose Dulbecco’s modified Eagle’s medium (DMEM, HyClone, Logan, UT, USA) supplemented with 10% FBS (Lonsera, URY), 100 U/mL penicillin (Procell, Wuhan, Hubei, China), and 100 µg/mL streptomycin (Procell, Wuhan, Hubei, China). SK-BR-3 cells were cultured in RPMI-1640 supplemented with 10% FBS, 100 U/mL penicillin, and 100 µg/mL streptomycin. Irinotecan, sunitinib, cisplatin, topotecan, gefitinib, exemestane, and idarubicin (purity >99%) were purchased from Selleck Chemicals (Houston, TX, USA). MTT and dimethyl sulfoxide (DMSO) were purchased from Sigma-Aldrich (St. Louis, MO, USA).

### 2.7. Cell Viability Assay

Cell viability was tested with the MTT assay [44]. MCF-7, MDA-MB-231, and SK-BR-3 cells were seeded in 96-well plates at 6 × 10^3^ cells/well for overnight incubation. The cells were exposed to irinotecan, sunitinib, cisplatin, topotecan, gefitinib, imatinib, and vorinostat alone or in the following combinations: irinotecan and sunitinib, exemestane and idarubicin, cisplatin and topotecan, or gefitinib and irinotecan. Seventy-two hours after drug treatment, 20 μL of a 5 mg/mL MTT solution was added to each well and incubated for another 4 h in a humidified atmosphere with 5% CO_2_ at 37 °C. Subsequently, the medium was removed, 150 μL of DMSO was added to each well, and plates were incubated for ten minutes. The resulting absorbance at a wavelength of 490 nm was detected by a fluorescence spectrometer (PerkinElmer EnVision, UK). By measuring the optical density and using a calibration curve, the growth inhibition rate was determined for each well. The 50% growth-inhibitory concentration (IC50) was calculated from fitted response curves. The ratios of drugs used in each combination were determined using the IC50 ratios of each drug. Each drug was applied in a concentration gradient, and the experiments were repeated for three biological replicates.

The interactions between two drugs were analysed according to the median effect principle proposed by Chou and Talalay [45]. The combination index (CI) and fraction affected (Fa) were calculated with CompuSyn software [46]. For the combination index, CI < 1, CI = 1, and CI > 1 represent synergism, additive effect, and antagonism, respectively. The Fa value represents the effect of the drug, that is, the tumour cell inhibition rate. The dose-reduction index (DRI) was used to measure the fold-reduction in a dose that may be produced by the synergistic combination of two drugs at a given effect level, as compared with treatment with each drug alone [47].

### 2.8. Wound Healing Assay

MCF-7 and MDA-MB-231 cells were seeded in 6-well culture plates at a density of 3 × 10^5^ cells/mL (2 mL/well) and grown to confluence, followed by drug treatment for 48 h in DMEM (the drug treatment groupings are shown in Table 1). The cell monolayer was scratched vertically with a sterile 10-µL micropipette tip. The detached cells were rinsed with phosphate-buffered saline (PBS) three times and then incubated in serum-free medium. Photographs of the central wound edges for each condition were taken at 0 h and 24 h. Two vertical lines were drawn on each side of the central wound. One line represented the location of faster-migrating cells and the other line represented the location of slower-migrating cells. The midpoint of the two vertical lines was the start or end of the calculation of the migration distance of the cells on that side. The cell migration rate was calculated as (0 h distance–24 h distance)/ 0 h distance.

### 2.9. In Vivo Cell Derived Xenograft Tumour Model

Female BALB/c nude mice (5 weeks old) were purchased from Beijing Vital River Laboratory Animal Technology Co., Ltd. (Beijing, China). MDA-MB-231 cells (1 × 10^7^) in 100 μL of PBS were injected into the back fat pad of 12 BALB/c nude mice. The tumour size was measured with callipers. The tumour volume was calculated as 0.5 × (longest measurement) × (shortest measurement)^2^. When the tumour diameter reached 2–3 cm, the tumours were harvested and placed in a sterile environment. The tumours were then cut into 1.5 mm × 1.5 mm uniform tumour blocks and inoculated separately into the backs of another 50 mice. After 1–2 weeks of inoculation, when the tumour volume reached 100–200 mm^3^, the tumour-bearing nude mice were randomly divided into groups according to the tumour volume. The experimental animals were divided into five groups with 10 mice in each group: glucose plus sorbitol aqueous solution as the blank control group; paclitaxel plus gemcitabine as an active control [48]; and gefitinib and irinotecan alone and combined as the experimental groups. The grouping and drug administration information are shown in Table 2. The doses of paclitaxel and gemcitabine were converted with reference to the actual usage in ClinicalTrials.gov (ClinicalTrials.gov Identifier: NCT01287624). The doses of gefitinib and irinotecan were based on the results of a preliminary dose tolerance experiment involving the administration of each drug alone. Tumour size and body weight were measured every other day for 14 days from the start of the drug treatment cycle. On day 14, the tumour size and mouse body weight were measured. Subsequently, the mice were sacrificed, and the subcutaneous tumours were harvested. The tumours were weighed and photographed. Three specimens were randomly selected from each group for haematoxylin-eosin (H&E, Procell, Wuhan, Hubei, China) staining. The Servicebio Institutional Animal Use Committee (IACUC) approved all of the experimental protocols (animal ethics permit NO. 2018006, approval date: 13 September 2018).

For histological analyses, tumour tissues were fixed in 4% paraformaldehyde and embedded in paraffin. Paraffin sections were stained with H&E for pathological analysis. Three tumour samples were taken from each group for staining.

### 2.10. Statistical Analysis of In Vivo and In Vitro Experiments

Statistical analysis of the wound healing assay and xenograft tumour model experiment were performed using GraphPad Prism 5.0 (GraphPad Software, San Diego, CA, USA). Data were documented as the mean ± standard deviation. One-way ANOVA (three or more groups) or two-tailed Student’s *t*-test (two groups) were employed to analyse the differences between groups. A *p*-value < 0.05 was considered statistically significant.

## 3. Results

### 3.1. Precision Drug Discovery for BC Individuals and Subtypes

**Figure 1:**
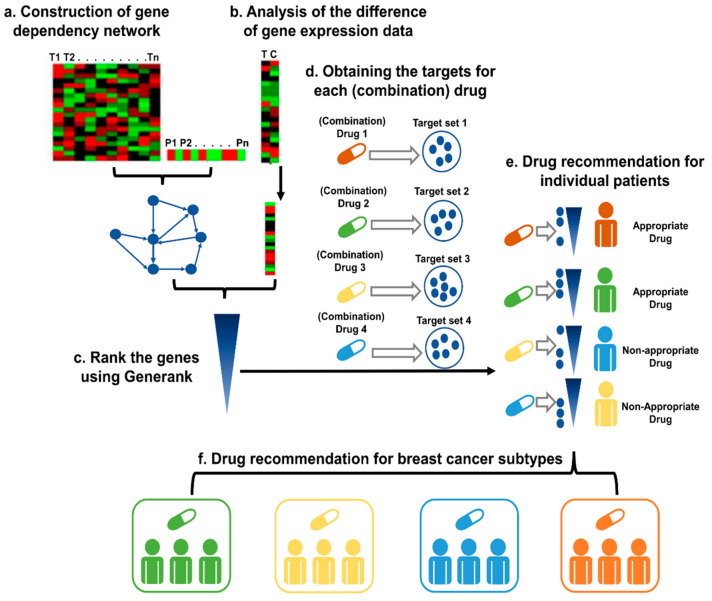
The pipeline for precision drug discovery. (**a**) Construction of the gene dependency network based on the gene expression data and prognostic information of the cancer patients. (**b**) The difference in gene expression levels between cancer tissue and control tissue, as indicated by the fold-change. (**c**) Based on the gene dependency network and the fold-changes of the genes of the patient, GeneRank was applied to rank the genes. (**d**) Identification of the targets for the candidate (combination) drugs. (**e**) For each drug, the Kolmogorov–Smirnov test was applied to test whether the targets were enriched among the top-ranked genes with our method. A drug is recommended as a suitable therapy if the drug targets the important genes of the patient. (**f**) The candidate (combination) drugs were defined as an appropriate therapy for a specific subtype if the effective rate in the subtype was significantly higher than that in all patients, which was evaluated with a binomial test.

The essence of medical therapy is using drugs to target disease-driven genes. Therefore, our precision drug discovery strategy was to identify the driver genes for cancer in each individual and screen personalized drugs by testing whether they could target the driver genes of the corresponding individual. The details of our strategy are shown in Figure 1.

First, based on the gene expression data and clinical information of the cancer patients, conditional mutual information was applied to identify the gene dependency network specific to the prognosis of cancer patients (see the Materials and Methods, Section 2.2). In this network, an edge from node A to node B indicates the mutual information of the expression level of gene A, and the prognostic risk of the cancer patient is significantly dependent on gene B. Thus, the genes with higher topological coefficients may be more important in cancer prognosis. For example, a gene with a higher out-degree indicates that the influence of more genes on the outcome of cancer patients would depend on it.

Second, for each patient, the difference in the gene expression levels between the cancer tissue and the control tissue was calculated as the fold-change. In this work, the expression level of the control was obtained by calculating the mean values of the healthy samples.

Based on the hypothesis that the importance of a gene may be evaluated by the importance of the genes that are dependent on it, a modified algorithm of GeneRank was applied to rank the genes for each sample. There were two inputs for the algorithm: the dependency network and the absolute values of the fold-change for all the genes in the network. The former input describes the dependency relations among all the genes, and the latter input indicates the initial importance of the genes. The importance of all the genes was calculated as the weighted summation of the importance of the genes that were dependent on it and the importance of the gene itself. The iteration of the algorithm stops when the importance of the genes converges. After that, we ranked all the genes for this sample according to the importance of these genes.

For each candidate drug or drug combination, the drug targets were collected from a well-known database (see the Materials and Methods, Section 2.4).

Finally, the effectiveness of the candidate drug or drug combination for a specific patient was predicted by testing whether the drug targeted the important genes of the patient. The Kolmogorov–Smirnov test was applied to test whether the drug targets were enriched among the top-ranked genes. A drug was considered a suitable therapy for the patient only if the drug targets were enriched among the key genes related to the prognosis of the patient.

Candidate (combination) drugs were defined as an appropriate therapy for a specific subtype if the effective rate in the subtype was significantly higher than that in all of the patients, which was evaluated by a binomial test.

Using our strategy, personalized drug (combination) treatment options could be prioritized for each patient. This strategy can also be used for drug combination discovery targeting different subtypes.

### 3.2. Precision Drug Discovery Strategy Evaluation

#### 3.2.1. Evaluation of the Gene Dependency Network

We presumed that the gene dependency network, which was reconstructed based on the gene expression data of a specific cancer, would reveal the gene dependencies related to the prognosis of this cancer. Therefore, the important genes in the network should be cancer related. We validated whether the important genes in the network were indeed cancer related by functional annotation.

We analysed the gene expression data for BC patients in TCGA, and the gene pairs with *p*-values less than 1 × 10^−5^, calculated with conditional mutual information, were selected to construct the gene dependency network related to the prognosis of BC (Table S2). To select the important genes in this network, we set the initial importance of all the nodes in the network as 1 and applied GeneRank to rank the genes in the network. All the genes were ranked based on their importance according to their topological locations in the network. In this work, the top 5% of genes (948 nodes) were selected as important genes in BC. Among these genes, 19 pathways with false discovery rates (FDRs) of less than 0.01 were identified as significantly enriched according to GSEA.

We found that ‘pathways in cancer’ was one of the most significant pathways (Table 3). Some subpathways, such as the ‘calcium signalling pathway’, ‘MAPK signalling pathway’, and ‘Wnt signalling pathway’, were also significantly enriched, among which, the calcium signalling pathway (FDR = 3.29 × 10^−7^) was the most significant; it was reported that curcumin induces the apoptosis of cancer cells through the calcium signalling pathway [49] and that the calcium signalling pathway may be a target for cancer treatment [50]. In summary, the important genes in the dependency network of BC were enriched for cancer-related pathways.

The gene dependency networks of OV (Table S3) and GBM (Table S4) were reconstructed based on their respective gene expression data. Applying the same strategy in BC, 890 genes were selected as important genes in the gene dependency network of OV. Sixteen pathways were significantly enriched by these genes (Table S5). Among these pathways, ‘DNA replication’ was the top-ranked, with an FDR of 5.98 × 10^−5^. DNA replication stress is a hallmark of cancer [51]. Furthermore, two immune-related pathways (the ‘T cell receptor signalling pathway’ and the ‘B cell receptor signalling pathway’) were also significantly enriched. In addition, some well-known cancer-related pathways, such as ‘focal adhesion’ (FDR = 3.35 × 10^−3^) and the ‘mTOR signalling pathway’ (5.68 × 10^−3^), were also enriched among our important genes, and focal adhesion kinase is reported to be a therapeutic target in OV [52]. Finally, ‘pathways in cancer’ were also significantly enriched with an FDR of 3.78 × 10^−3^, which may indicate that the important genes in the gene dependency network of OV are indeed cancer-related.

The gene dependency network of GBM prioritised 890 genes, and 21 pathways were significantly enriched for these genes (Table S6). Dysregulation of the actin cytoskeleton [53] and cell adhesion [54,55] pathways is related to cancer cell metastasis. ‘Regulation of the actin cytoskeleton’ (1.15 × 10^−6^), ‘focal adhesion’ (1.15 × 10^−6^), and ‘adherens junction’ (8.14 × 10^−6^) were the most significantly enriched pathways. Some subpathways in the ‘pathways in cancer’, such as the chemokine signalling pathway, Wnt signalling pathway, and MAPK signalling pathway, were significantly enriched. ‘Glioma’ was also significantly enriched, with an FDR of 2.68 × 10^−3^.

These results demonstrate that the gene dependency network may be used to reveal the gene dependencies of genes related to cancer prognosis. Therefore, this network may facilitate the identification of genes that are important in cancer prognosis.

#### 3.2.2. Evaluation of GeneRank Prioritization

As the gene dependency network could reveal the gene dependencies in cancer prognosis, and the fold-change in the gene expression levels between the disease tissue and control tissue could describe the differential expression of genes in a specific patient, GeneRank, which takes both the gene dependency network and the initial importance of the nodes (the fold-change in the gene expression levels between the disease tissue and control tissue) as input, can be used to prioritize the genes related to the prognosis of a specific patient. To validate our strategy, we ranked the genes for each patient (including BC, OV, and GBM patients), and the Kolmogorov–Smirnov test was applied to determine whether the cancer genes were enriched among the top-ranked genes for each patient. For comparison, the same number of random gene lists were applied to perform the same analysis.

**Figure 2:**
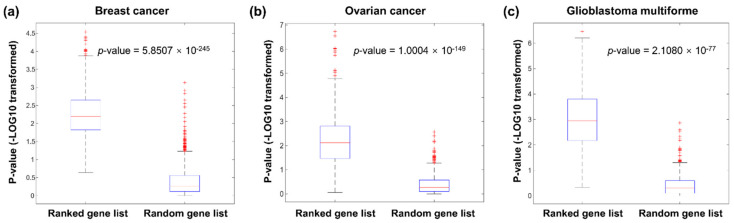
Analysis of the enrichment of known cancer genes among the ranked gene lists of all assessed BC (**a**), OV (**b**), and GBM (**c**) patients.

Among the 778 BC samples, the ranked gene lists of 93.44% of the samples were significantly enriched in genes related to BC. However, only 3.60% of the genes in the random gene lists were significantly enriched. The Mann–Whitney U test showed that the ranked gene lists derived from our method were significantly more enriched for BC-related genes than the random gene lists, with a *p*-value of 5.8507 × 10^−245^ (Figure 2a).

OV-related genes were significantly enriched in 79.96% of the ranked gene lists. However, only 4.54% of the random gene lists were significantly enriched in these cancer genes. The *p*-value of the Mann–Whiney U test comparing the enrichment *p*-values of the ranked genes with our method and the random gene lists was 1.0004 × 10^−149^ (Figure 2b).

A similar result was found in GBM. Our method prioritised the known cancer genes of GBM in 94.31% of patients, and this ratio was 5.69% in the control group. The *p*-value of the Mann–Whiney U test comparing the enrichment results of the two groups was 2.1080 × 10^−77^ (Figure 2c). These results demonstrate that our method may be used to identify driver genes in individual patients, which supports the effectiveness of our strategy for personalised drug discovery.

#### 3.2.3. Evaluation of Precision Drug Discovery

As demonstrated above, our method may be used to identify important genes for each patient based on the gene expression profile of the corresponding patient. Therefore, it is reasonable to expect drug therapy to be effective only when the important genes match the drug targets. The present study used the Kolmogorov–Smirnov test for target enrichment evaluation, and drugs (or drug combinations) that targeted the top genes of the ranked gene list using our method were considered suitable for the patient (precision drug discovery). For each patient, all drugs (or drug combinations) could be ranked from most to least suitable, according to the *p*-value of the Kolmogorov–Smirnov test in ascending order.

This strategy was primarily validated using OV and GBM data derived from TCGA, which documents drug treatment and prognosis information for a large number of cancer patients. The effects of the drugs in each patient were reflected by the Kolmogorov–Smirnov *p*-values. When a patient was treated with more than one drug, the effect for the patient was set as the best drug used. Patients with each type of cancer were divided into two groups of the same size based on the drug effect in these patients. Patients with Kolmogorov–Smirnov *p*-values below the median were included in the properly treated group; otherwise, the patients were included in the improperly treated group.

**Figure 3:**
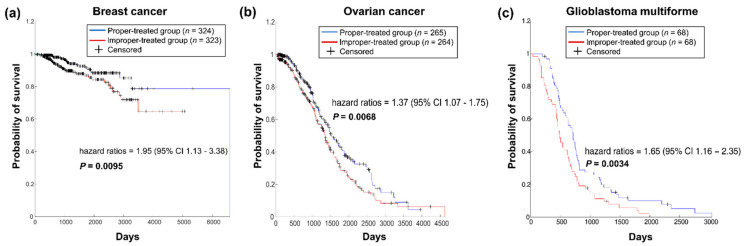
Survival analysis of the two patient groups divided by administration of the appropriate therapy. (**a**). Survival analysis of BC patients. (**b**). Survival analysis of OV patients. (**c**). Survival analysis of GBM cancer patients.

A total of 324 BC patients were considered properly treated, and 323 patients were considered improperly treated. The survival analysis showed that the hazard ratio of the two groups was 1.95 (95% CI 1.13–3.38), and the log-rank *p*-value was 0.0095 (Figure 3a). The hazard ratio between the 265 properly treated OV patients and the 264 improperly treated patients was 1.37 (95% CI 1.07–1.75), and the log-rank *p*-value was 0.0068 (Figure 3b). Our method also distinguished the prognostic risk of GBM patients (Figure 3c). The hazard ratio of the two groups of GBM patients was 1.65 (95% CI 1.16–2.35), and the *p*-value of the log-rank test was 0.0034.

The cancer patients in all three data sets who were properly treated with drugs, as predicted by our method, had longer survival times than patients who were improperly treated, which may indicate that our method prioritised the appropriate drugs for individual patients.

### 3.3. Precision Drug Discovery for BC Subtypes

BC patients are divided into four major molecular subtypes: luminal A (ER+, PR±, HER2−), luminal B (ER+, PR±, HER2±), HER2+ (ER−, PR−, HER2+), and TNBC (ER−, PR−, HER2−) [56]. One of the main purposes of the present study was to predict drug effects in patients with specific subtypes of cancer. Therefore, we also used our method to screen drug combinations in specific subtypes of BC. The TCGA provides clinical information of three indicators—ER, PR, and HER2—for BC patients. We used these three indicators to divide all BC patients into luminal A (ER+, PR±, HER2−), luminal B (ER+, PR±, HER2+), HER2+ (ER−, PR−, HER2+), and TNBC (ER−, PR−, HER2−) subtypes, and these groups included 373, 99, 37, and 116 patients, respectively. Based on drug combinations with confirmed anticancer activity in phase II and higher trials, according to ClinicalTrials.gov data from the DCDB, a binomial test was used to predict whether drug combinations were significantly effective in a certain BC subtype. The drug combinations for specific subtypes of BC as predicted in this study are shown in Table S7. Fourteen drug combinations with positive clinical trial results were selected from the above prediction results. Interestingly, 11 pairs of drugs that we predicted to be effective in specific subtypes had clinical response data that were consistent with our prediction (Table 4), while the remaining 3 predicted drug combinations were invalidated by inconsistent clinical trial results (Table 5).

From the above results, the predictive accuracy of this method could be as high as 78.6% (11/14), and thus our strategy could be used to identify drugs for patients with specific subtypes of cancer.

### 3.4. In Vitro Experimental Validation of Drug Combinations

We validated the drugs predicted for specific subtypes of BC with cell-based experiments. The model predicted that four drug combinations, which were not reported as related to BC activity (i.e., gefitinib and irinotecan, irinotecan and sunitinib, cisplatin and topotecan, and exemestane and idarubicin), would be efficacious (*p*-value < 0.05) in BC of the TNBC subtype (Table 6).

We selected three cell lines, MCF-7 (luminal A), MDA-MB-231 (TNBC), and SK-BR-3 (HER2+), to represent different BC subtypes for drug activity verification. The dose–effect relationships of irinotecan, sunitinib, cisplatin, topotecan, gefitinib, exemestane, and idarubicin alone or in combination on the growth of human BC cells (MCF-7, MDA-MB-231, and SK-BR-3) were evaluated after 72 h of treatment. The median-effect analysis was completed according to the method of Chou [47].

These drugs showed dose-dependent cytotoxic effects in MCF-7, MDA-MB-231, and SK-BR-3 cells (Figures S1–S3), and the IC50 values of these drugs are shown in Table 7. As the Chou-Talalay model requires fixed dose ratios, the ratios of these drug combination pairs were set based on the IC50 values for each cell line (Table 8, Table 9, Table 10 and Table 11).

**Figure 4:**
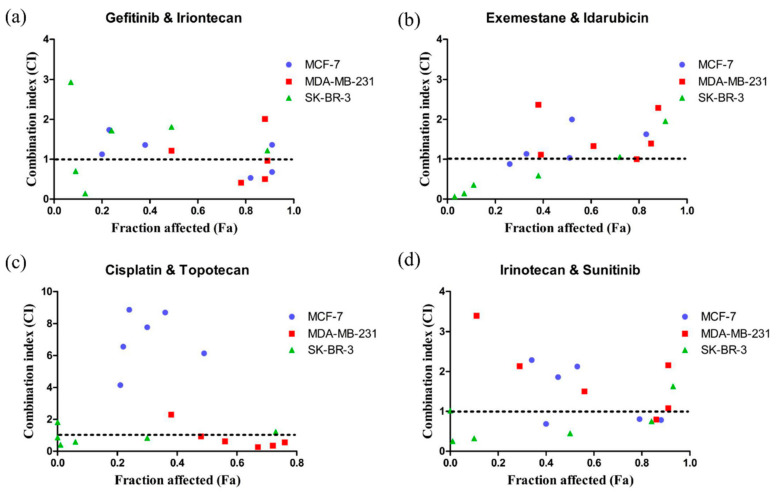
Scatter plots of CI versus Fa. (**a**) gefitinib combined with irinotecan. (**b**) exemestane combined with idarubicin. (**c**) cisplatin combined with topotecan. (**d**) irinotecan combined with sunitinib. The synergistic effect of the combination of gefitinib and irinotecan on MCF-7, MDA-MB-231, and SK-BR-3 cells can be seen. The CI value and fraction affected (Fa) was calculated via CompuSyn software (ComboSyn, Inc., Paramus, NJ. 07652 USA). CI < 1 indicates a synergistic effect, CI = 1 indicates an additive effect, and CI > 1 indicates an antagonistic effect. Fa value represents the effect of the drug, that is, the tumour cell inhibition rate.

The antitumour effect of the drug combinations was determined according to the CI. The effects of these drug-pair combinations on cell growth are shown in Table 8, Table 9, Table 10 and Table 11 and Figure 4. CI values of <1, 1, and >1 indicate a supra-additive effect (synergism), additive effect, and an antagonistic effect, respectively. Fa value represents the effect of the drug, that is, the tumour cell inhibition rate.

A lower CI value was observed for the combination of gefitinib and irinotecan in the MCF-7 and MDA-MB-231 cell lines when the Fa value was >0.6 (Figure 4a). Similar to the effects of gefitinib and irinotecan, synergistic effects were also observed in MDA-MB-231 cells over a wide range of inhibition levels for the combination of cisplatin and topotecan (Figure 4c). The combination of cisplatin and topotecan had an antagonistic effect in MCF-7 cells and lower inhibition levels in SK-BR-3 cells (Figure 4c). For the combination of exemestane and idarubicin, an additive effect (CI = 0.99996) was observed in MDA-MB-231 cells (Figure 4b). The combination of irinotecan and sunitinib had a similar synergistic effect in all three cell lines when the Fa value was greater than 0.6.

Among the four drug combinations we predicted with anti-TNBC activity, three sets of prediction results for TNBC were consistent with the results of MTT experimental verification, and these three drug combinations had a synergistic effect on TNBC cell lines in vitro. According to records from the Drug Combination Database Version 2.0 (DCDB 2.0, http://www.cls.zju.edu.cn/dcdb/, accessed on 18 May 2018), only 3.9% (53/1363) of included drug combinations have clinical activities against breast cancer [57]. However, the results of MTT experiments showed that three of the four predicted drug combinations had anti-TNBC activities, which is significantly higher than the ratio from the DCDB.

**Figure 5:**
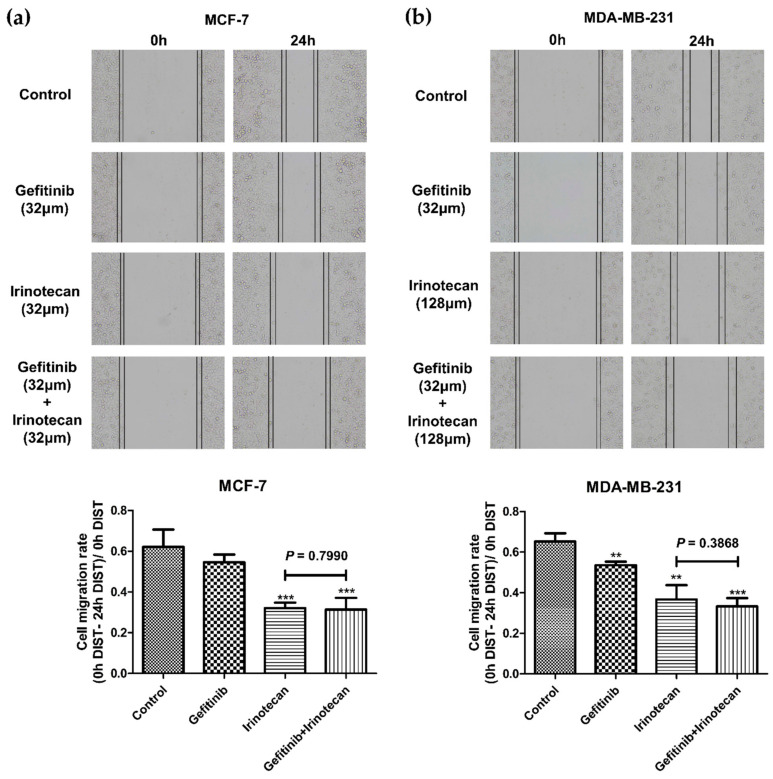
Effects of gefitinib and irinotecan on the migration of BC cell lines. The cell migration rate was calculated as (0 h distance–24 h distance)/ 0 h distance. (**a**) In the MCF-7 cell line, the cell migration rates of the gefitinib, irinotecan, and gefitinib plus irinotecan groups were significantly lower than that of the control group at 24 h, while there was no significant difference between the gefitinib plus irinotecan group and the irinotecan group. (**b**) In the MDA-MB-231 cell line, the cell migration rates of the gefitinib, irinotecan, and gefitinib plus irinotecan groups were significantly lower than that of the control group at 24 h, and the migration rate of the gefitinib plus irinotecan group was significantly lower than that of the irinotecan group. * *p* < 0.05, ** *p* < 0.01, and *** *p* < 0.001, as compared with the control group.

Because the combination of gefitinib and irinotecan had a higher Fa value and stronger synergistic effect, we selected this drug combination for further activity testing. The MTT results suggested that the combination of gefitinib and irinotecan exhibited synergistic effects on MCF-7 and MDA-MB-231 cells but not on SK-BR-3 cells. Therefore, we used a wound healing assay to further test the combined effect of gefitinib and irinotecan on cell migration in the MCF-7 and MDA-MB-231 cell lines. As shown in Figure 5, the MDA-MD-231 cell line showed stronger cell migration ability than the MCF-7 cell line, which was consistent with existing studies. The combination of gefitinib and irinotecan showed a significant ability to restrain cell migration in both cell lines. However, the single drug irinotecan showed the same ability as the combination in the MCF-7 cell line. Therefore, this combination was not synergistic. In contrast, the combination of gefitinib and irinotecan in the MDA-MB-231 cell line performed significantly better than these two drugs alone in terms of inhibiting cell migration.

Briefly, the combination of gefitinib and irinotecan had a significant synergistic effect on the proliferation and migration of MDA-MB-231 cells. We further validated the inhibitory effect of this combination on a TNBC subtype cell lines (MDA-MB-231) via a cell-derived xenograft model (CDX).

### 3.5. In Vivo Combination Effects of Gefitinib and Irinotecan

**Figure 6:**
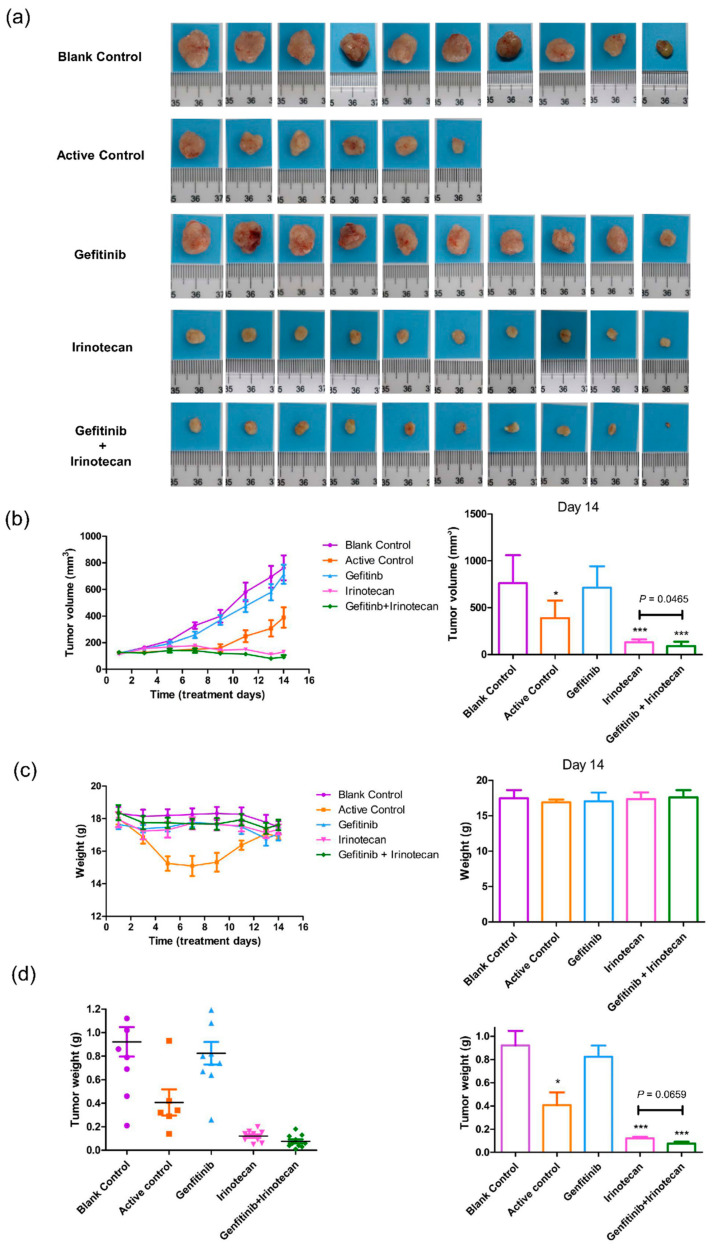
Effects of gefitinib and irinotecan on cell-derived xenograft models constructed with MDA-MB-231 cell lines. (**a**) After 14 days of drug treatment, the tumours of the nude mice were removed and photographed on a 2 cm × 2 cm light blue cardboard platform (ten nude mice in each group; four nude mice in the active control group died; therefore, four pictures are missing from (a)). (**b**) Trends in tumour volume in each drug treatment group during the 14-day dosing cycle (measured with Vernier callipers). At the end of the 14th day, the tumour volumes of the active control (paclitaxel plus gemcitabine), irinotecan, and gefitinib plus irinotecan groups were significantly smaller than that of the blank control group, and the tumour volume of the gefitinib plus irinotecan group was significantly smaller than that of the irinotecan group. (**c**) The positive control group continued to decrease in weight five days after administration, indicating that the drug combination was highly toxic to nude mice according to this metric and resulting in intolerance. After ceasing the administration of gemcitabine on the sixth day, the weight of the surviving mice in this group gradually recovered. The other groups of mice remained stable during the 14-day treatment period. At the end of the 14th day, there was no significant difference in body weight between the groups. (**d**) On the 14th day, the subcutaneous tumours of all nude mice were removed for weighing. The tumour weights of the active control, irinotecan, and gefitinib plus irinotecan groups were significantly lower than those of the blank control group. The tumour weight of the gefitinib plus irinotecan group was lower than that of the irinotecan group, and the *p* value was 0.0659, which indicated marginal significance. * *p* < 0.05, and *** *p* < 0.001, as compared with the blank control group.

In vivo experiments were designed to evaluate the combined therapeutic effect of oral gefitinib and intraperitoneal irinotecan on the TNBC subtype of BC. The growth-inhibitory effect of the combination was evaluated in tumour xenografts derived from TNBC subtype cells. No growth-inhibitory effects were observed when gefitinib alone was applied to animals xenografted with MDA-MB-231 cells, and the results were similar to those of the blank control group; the average tumour volume reached 600 mm^3^ or more (Figure 6a,b). Administration of paclitaxel combined with gemcitabine (active control), single-agent irinotecan, and gefitinib combined with irinotecan significantly restricted tumour growth during the first five days (Figure 6b). However, paclitaxel combined with gemcitabine was more toxic to nude mice, and all nude mice showed a sharp decrease in body weight. On the fifth day of treatment, four nude mice died in the active control group (Figure 6a,c). Therefore, gemcitabine administration in the active group was stopped on the sixth day, but paclitaxel administration was continued. Thereafter, the active control group showed a poor tumour-suppressing effect similar to that of the single gefitinib treatment group, and the lost body weight was recovered by day 14 (Figure 6b,c).

Unlike the active control group, the single-agent irinotecan and gefitinib combined with irinotecan drug treatment groups maintained good tumour growth inhibition activity (Figure 6a,b). After the sixth day of administration, the tumours of nude mice displayed a shrinking trend, and the difference in tumour size was significant compared with that of the blank control group; the *p*-value was less than 0.001 at day 14 (Figure 6a,b,d). A more marked growth-inhibitory effect was observed with gefitinib combined with irinotecan. On day 14, the tumour volume of the gefitinib combined with irinotecan group was significantly lower than that of the single-agent irinotecan group (*p*-value = 0.0465), and the tumour weight was also lower than that of the single-agent irinotecan group (*p*-value = 0.0659) (Figure 6a,b,d). Except for the active control group, the weight of the nude mice in the other treatment groups remained stable during the 14-day treatment period, indicating that the nude mice were tolerant to the doses of gefitinib and irinotecan in this experiment (Figure 6c). No deaths were observed during the treatment or observation period except in the active control group.

These results indicate that the combination of gefitinib and irinotecan has a significant synergistic effect in the treatment of TNBC subtype cells. Additionally, the combination has higher activity and lower toxicity than the known anti-TNBC subtype combination of paclitaxel and gemcitabine.

### 3.6. Combination Effects of Gefitinib and Irinotecan on Tumour Cell Apoptosis

**Figure 7:**
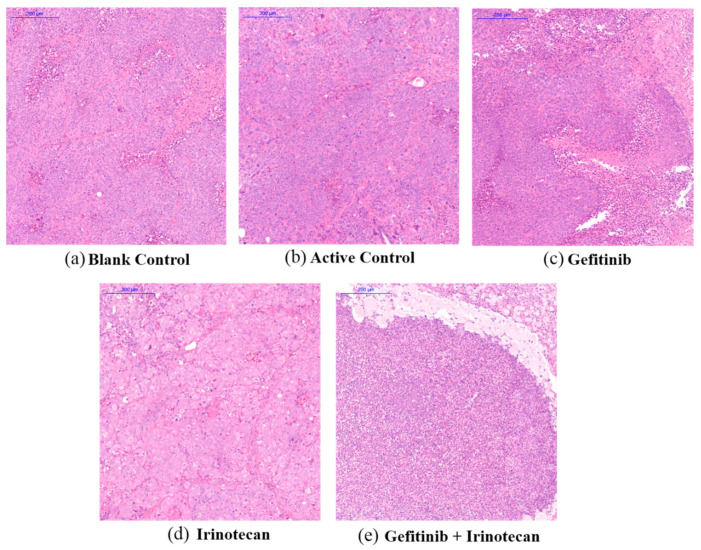
Effect of drug treatment on the pathological changes of transplanted tumours in nude mice, as indicated by H&E staining. (**a**,**b**) The solvent blank control group and the active control group of paclitaxel plus gemcitabine, respectively. (**c**,**d**) The single-agent gefitinib group and the single-agent irinotecan group, respectively. (**e**) Gefitinib plus irinotecan combination group. The scale bar of the subfigures is 200 μm.

To evaluate the effect of gefitinib and irinotecan on the pathological changes of tumour cells, we performed H&E staining analysis of the tumour tissue after CDX drug treatment. According to the H&E staining of the pathological sections, the blank control group had larger tumour cells; blue-violet, large, and uniform nuclei; and mitotic figures (Figure 7a). The tumour cells of the paclitaxel combined with gemcitabine and gefitinib groups were similar to those of the control group. The tumour cells were in a state of nuclear fission; however, some tumour cells died in the gefitinib group (Figure 7b,c). Larger tumour cells were seen in the irinotecan group. In this group, the nuclei were smaller, the staining was lighter, the ratio of the nucleus to the nucleus was slightly larger, and the cells were not in a proliferative state (Figure 7d). Most of the tumour cells were necrotic, and the nuclei were concentrated and deformed in the gefitinib plus irinotecan group (Figure 7e). These results indicate that gefitinib combined with irinotecan can not only block division of TNBC subtype cells, but can also promote cell lysis and death. These pathological phenomena support the results of the previous in vivo experiments.

## 4. Discussion

BC is the most common female cancer. It is a heterogeneous disease, and different patients benefit from different treatments. Therefore, it is essential to screen drugs for individuals. Most current personalized therapy strategies depend on biomedical big data and statistically derived information on genetic variation. However, this paradigm faces some fundamental difficulties in practice. First, the penetrance for the variants is generally not known, which hinders therapeutic decision-making. Second, the prediction models established using statistical or machine-learning methods are sensitive to the data sets used, resulting in poor generalisation capability. The essence of medical therapy is the use of drugs to act on disease-driven genes, also termed targets. Therefore, the identification of driver genes in each patient could easily prioritise a suitable drug that would target these identified driver genes. Many genes work together to affect the prognosis of complex diseases (e.g., BC). The present study constructed a gene dependency network to reveal the gene dependency relationships between genes in the cancer prognosis of BC. To validate our gene dependency network, the genes with the top out-degrees were selected. Functional annotation showed that these genes were primarily associated with cancer-related pathways. We used a gene dependency network and the fold-change in the gene expression levels between disease tissue and control as inputs, and the GeneRank algorithm was used to prioritize the essential genes for patients. Using the driver genes from GeneCards, enrichment analysis demonstrated that our strategy identified the driver genes for each patient. The Kolmogorov–Smirnov test was used to determine whether the drug targets were enriched among the top-ranked genes for each patient to evaluate drugs for the individual patient. Survival analysis on BC data sets in TCGA showed that the patients with suitable treatments had longer survival times than the other patients, which validated our personalised therapy strategy. Our strategy was also validated in OV and GBM in TCGA. The effective results in OV and GBM show the robustness of our strategy to a certain extent.

BC patients are divisible into four subtypes. TNBC is the more aggressive subtype, and it has fewer treatment options. Drug combination therapies would improve the outcomes of TNBC. Therefore, it is urgent to screen drug combinations for TNBC. We used our strategy to screen drug combinations for each of the BC patients, and drug combinations with higher efficiencies in TNBC patients compared with the other patients were selected as candidates for this subtype. We selected three cell lines, MCF-7, MDA-MB-231, and SK-BR-3, which represent different BC subtypes, to verify the drug activity of four pairs of candidate drug combinations. The results of the MTT assay suggested that all four drug combinations had synergistic effects on TNBC cell lines (MDA-MB-231) in vitro, which was consistent with the theoretical predictions. Among the four pairs of candidate drug combinations, gefitinib and irinotecan demonstrated a better synergistic inhibitory effect in the TNBC cell line than the others did. This drug combination also showed a good synergistic effect on the MCF-7 cell line. Therefore, the ability of this drug combination to inhibit the migration of MCF-7 and TNBC cells was further tested. The results of the wound healing assay showed that gefitinib and irinotecan had a significant inhibitory effect on the migration of TNBC cells. We used a xenograft model to test the in vivo activity of gefitinib and irinotecan. The experimental results also demonstrated that the combination of gefitinib and irinotecan had a significant synergistic effect in the treatment of TNBC subtype cells. The combination also had higher activity and lower toxicity than the known anti-TNBC subtype combination of paclitaxel and gemcitabine.

Although we proposed a rational strategy for precision drug discovery, there are some drawbacks in our strategy. First, we constructed a gene dependency network of BC to reveal the gene dependency relationship in the prognosis of cancer patients and used it to rank the essential genes for each patient. However, the gene dependency relationship may vary between patients. Therefore, the drug screening method may be improved if we could construct a personalized network for each patient. Second, we used gene expression data to identify driver genes for each patient because it could reveal genomic changes, environmental changes, and other factors. However, other data types, such as genomic and epigenetic data, may also be useful for application. Our strategy cannot screen drugs with unknown target information. Therefore, the use of other data sets, such as the drug-stimulating data from LINCS1000 [58] to infer essential genes in drug responses and use them as the ‘target gene’ in our pipeline, would expand the scope of application of our method.

## 5. Conclusions

Our research combined the GeneRank algorithm and a gene dependency network and proposed a systems pharmacology-based drug combination discovery strategy. The gene dependency network related to the prognosis of BC patients was used as the biological network input for the GeneRank algorithm, and a patient’s gene expression data were used as the input for that patient’s important nodes in the gene dependency network. The patient’s genes were ranked by importance. Therefore, it was possible to test whether the candidate drug (or drug combination) targets were significantly enriched in the patient’s important genes to determine the effectiveness of the candidate drugs for the patient and establish accurate medication recommendations.

We identified all of the drug combinations with established anticancer activity in phase II and higher trials that were recorded at ClinicalTrials.gov and collected them from the DCDB. We used the gene expression data of patients with BC subtype information in the TCGA database and established an accurate drug screening approach for different BC subtypes. The drug combinations with anticancer activity that were recorded in clinical phase II or higher trials were obtained from the DCDB as candidate drug combinations for drug subtype prediction. We screened drug combinations with active clinical subtype records for BC, compared the clinical results with the predicted results, and verified the accuracy of the strategy at a theoretical level.

We used this strategy to screen four pairs of drug combinations in different BC subtypes. In vivo and in vitro experiments verified that the combination of gefitinib and irinotecan inhibited the proliferation and migration of TNBC cells.

Collectively, these results indicate, at the theoretical and experimental levels, that the presented precision drug discovery strategy effectively identified important disease-related genes in individuals or special groups to achieve precise and efficient drug discovery. This strategy has the advantages of easy implementation, high efficiency, low economic cost, and strong operability. It may be widely used in precision medicine for specific patients and patient groups, and it is helpful for providing theoretical guidance for drug development for specific patient groups.

## Figures and Tables

**Table 1:** Group information of wound healing analysis.

Group	MCF-7 (Drug Treatment)	MDA-MB-231 (Drug Treatment)
Control	Solvent	Solvent
Gefitinib	Gefitinib (32 μM)	Gefitinib (32 μM)
Irinotecan	Irinotecan (32 μM)	Irinotecan (128 μM)
Gefitinib + Irinotecan	Gefitinib (32 μM) +Irinotecan (32 μM)	Gefitinib (32 μM) +Irinotecan (128 μM)

**Table 2:** Grouping of xenotransplantation experiments and drug treatment information.

Groups	Number	Drug Treatment (Dosage of Administration; Administration Route; Delivery Days)
Blank Control	1–10	5% glucose solution (the dose volume was approximately equal to group Gefitinib plus Irinotecan)
Active Control	11–20	Paclitaxel (20 mg/kg; ip ^a^; 1,8); Gemcitabine (35 mg/kg, ip, 1,2,3,14)
Gefitinib	21–30	Gefitinib (30 mg/kg; ig ^b^; 1,3,5,7,9,11,13)
Irinotecan	31–40	Irinotecan (8 mg/kg; ip; 1,3,5,7,9,11,13)
Gefitinib + Irinotecan	41–50	Gefitinib (30 mg/kg; ig; 1,3,5,7,9,11,13), Irinotecan (8 mg/kg; ip; 1,3,5,7,9,11,13)

^a^ intraperitoneal injection; ^b^ gavage.

**Table 3:** The enriched pathways of important genes in BC (FDR q-value < 0.01).

Gene Set Name	*p*-Value	FDR q-Value
Calcium signalling pathway	1.77 × 10^−9^	3.29 × 10^−7^
Pathways in cancer	2.72 × 10^−8^	2.53 × 10^−6^
MAPK signalling pathway	2.65 × 10^−7^	1.64 × 10^−5^
Focal adhesion	3.79 × 10^−7^	1.76 × 10^−5^
Neuroactive ligand–receptor interaction	5.95 × 10^−6^	2.21 × 10^−4^
Wnt signalling pathway	7.19 × 10^−6^	2.23 × 10^−4^
Vascular smooth muscle contraction	1.33 × 10^−5^	3.55 × 10^−4^
Gap junction	5.68 × 10^−5^	1.22 × 10^−3^
Purine metabolism	5.91 × 10^−5^	1.22 × 10^−3^
Lysosome	1.14 × 10^−4^	2.11 × 10^−3^
Melanogenesis	1.50 × 10^−4^	2.54 × 10^−3^
Small cell lung cancer	2.05 × 10^−4^	3.18 × 10^−3^
Oocyte meiosis	3.48 × 10^−4^	4.98 × 10^−3^
Fructose and mannose metabolism	4.38 × 10^−4^	5.81 × 10^−3^
Glycosaminoglycan degradation	6.06 × 10^−4^	6.83 × 10^−3^
Basal cell carcinoma	6.17 × 10^−4^	6.83 × 10^−3^
Phosphatidylinositol signalling system	6.24 × 10^−4^	6.83 × 10^−3^
Hedgehog signalling pathway	6.81 × 10^−4^	7.03 × 10^−3^
Regulation of actin cytoskeleton	9.83 × 10^−4^	9.63 × 10^−3^

**Table 4:** Actual and predictive activity of BC drugs (consistent).

DCDB ID	Drug Combination	Predicted Subtypes	Clinical Trial Subtypes	Clinical Effectiveness
DC000083	arzoxifene + LG100268	TNBC/HER2+	TNBC/HER2+	efficacious
DC000132	paclitaxel + trastuzumab	HER2+/Luminal B	HER2+/Luminal B	efficacious
DC000225	gemcitabine + trastuzumab	HER2+	HER2+/Luminal B	efficacious
DC000227	epirubicin + trastuzumab	HER2+	HER2+/Luminal B	efficacious
DC000231	trastuzumab + vinorelbine	HER2+	HER2+/Luminal B	efficacious
DC000233	cyclophosphamide + trastuzumab	HER2+/Luminal B	HER2+/Luminal B	efficacious
DC000236	carboplatin + trastuzumab	HER2+	HER2+/Luminal B	efficacious
DC000649	lapatinib + paclitaxel	HER2+/Luminal B	HER2+/Luminal B	efficacious
DC006857	cediranib + olaparib	TNBC	TNBC/HER2+	efficacious
DC001856	anastrozole + gefitinib	TNBC/HER2+	Luminal A/Luminal B	non-efficacious
DC002772	fulvestrant + gefitinib	TNBC/HER2+	Luminal A/Luminal B	non-efficacious

**Table 5:** Actual and predictive activity of BC drugs (inconsistent).

DCDB ID	Drug Combination	Predicted Subtypes	Clinical Trial Subtypes	Clinical Effectiveness
DC000089	gefitinib + trastuzumab	HER2+	HER2+	non-efficacious
DC000220	lapatinib + letrozole	TNBC/HER2+	Luminal A/Luminal B	efficacious
DC000229	doxorubicin + trastuzumab	Luminal A	HER2+	efficacious

**Table 6:** Subtype prediction results for four drug combinations (*p*-values evaluated with binomial tests).

Drug Combination	Luminal A	Luminal B	HER2+	TNBC
gefitinib + irinotecan	0.9233	0.2787	0.1644	0.0401
exemestane + idarubicin	0.9998	0.0819	0.0241	7.9100 × 10^−11^
cisplatin + topotecan	1.0000	0.6619	<1 × 10^−16^	<1 × 10^−16^
irinotecan + sunitinib	0.9233	0.6619	0.5039	0.0006

**Table 7:** Single drug IC50 value determination.

Drug Name	MCF-7		MDA-MB-231		SK-BR-3	
	IC50 (µM)	Concentration Range (µM)	IC50 (µM)	Concentration Range (µM)	IC50 (µM)	Concentration Range (µM)
Gefitinib	35.19	4.69–600	55.20	4.69–600	5.55	0.00768–600
Irinotecan	34.76	0.002–150	201.27	0.1–300	26.36	0.00768–600
Exemestane	145.78	4.688–600	127.37	4.688–600	128.38	0.00768–600
Idarubicin	0.31	0.00256–200	0.26	0.00256–200	0.02	0.00768–600
Cisplatin	47.12	2.34–300	47.12	2.34–300	8.46	0.00768–600
Topotecan	0.48	0.1–300	103.57	0.1–300	0.74	0.00768–600
Sunitinib	4.05	0.1–300	8.37	0.1–300	5.94	0.00768–600

**Table 8:** Fa and CI values for actual experimental points of gefitinib combined with irinotecan.

MCF-7 (1:1) ^a^			MDA-MB-231 (1:4) ^b^			SK-BR-3 (0.03:0.16) ^c^		
Total Dose	Fa	CI Value	Total Dose	Fa	CI Value	Total Dose	Fa	CI Value
256.00	0.91	1.36100	640.00	0.88	2.01029	93.00	0.89	1.22031
128.00	0.91	0.68050	320.00	0.89	0.96101	31.00	0.49	1.81269
64.00	0.82	0.53076	160.00	0.88	0.50257	10.34	0.24	1.71940
32.00	0.38	1.35817	80.00	0.78	0.41251	3.45	0.07	2.93259
16.00	0.23	1.73336	40.00	0.49	1.21187	1.15	0.09	0.70258
8.00	0.20	1.12579				0.38	0.13	0.14314

^a b c^ The dose ratio of gefitinib and irinotecan in each cell line. The ratio of the drug combination was set according to the IC50 value of the single drug in each cell line.

**Table 9:** Fa and CI values for actual experimental points of exemestane combined with idarubicin.

MCF-7 (4.69:0.01) ^a^			MDA-MB-231 (4.69:0.01) ^b^			SK-BR-3 (1.6:2.0E-4) ^c^		
Total Dose	Fa	CI Value	Total Dose	Fa	CI Value	Total Dose	Fa	CI Value
300.60	0.83	1.62448	300.60	0.88	2.28575	390.06	0.91	1.95615
150.30	0.52	1.99761	150.30	0.85	1.39243	130.02	0.72	1.05564
75.15	0.51	1.03379	75.15	0.79	0.99996	43.34	0.38	0.59039
37.58	0.33	1.13269	37.58	0.61	1.32759	14.44	0.11	0.35679
18.79	0.26	0.87867	18.79	0.38	2.36631	4.81	0.07	0.14347
9.40	0.05	9.02758	9.40	0.39	1.11416	1.60	0.03	0.06721

^a b c^ The dose ratio of exemestane and idarubicin in each cell line. The ratio of the drug combination was set according to the IC50 value of the single drug in each cell line.

**Table 10:** Fa and CI values for actual experimental points of cisplatin combined with topotecan.

MCF-7 (3.13:0.03) ^a^			MDA-MB-231 (0.41:1.23) ^b^			SK-BR-3 (0.12:0.01) ^c^		
Total Dose	Fa	CI Value	Total Dose	Fa	CI Value	Total Dose	Fa	CI Value
100.80	0.49	6.13662	400.00	0.76	0.55871	32.25	0.73	1.22184
50.40	0.36	8.68534	133.33	0.72	0.34791	10.75	0.30	0.83846
25.20	0.30	7.77286	44.44	0.67	0.26373	3.58	0.06	0.59108
12.60	0.24	8.85453	14.81	0.56	0.62049	1.19	0.01	0.40758
6.30	0.22	6.55762	4.93	0.48	0.93238	0.40	1.0E-4	0.86656
3.16	0.21	4.14541	1.64	0.38	2.29294	0.13	1.0E-6	1.82670

^a b c^ The dose ratio of cisplatin and topotecan in each cell line. The ratio of the drug combination was set according to the IC50 value of the single drug in each cell line.

**Table 11:** Fa and CI values for actual experimental points of irinotecan combined with sunitinib.

MCF-7 (3.13:0.03) ^a^			MDA-MB-231 (0.41:1.23) ^b^			SK-BR-3 (0.12:0.01) ^c^		
Total Dose	Fa	CI Value	Total Dose	Fa	CI Value	Total Dose	Fa	CI Value
114.80	0.88	0.78207	537.60	0.91	2.15513	96.00	0.93	1.62976
57.40	0.79	0.80721	268.80	0.91	1.07757	32.00	0.84	0.75227
28.70	0.53	2.12400	134.40	0.86	0.79456	10.67	0.50	0.44974
14.35	0.45	1.85993	67.20	0.56	1.49972	3.56	0.10	0.32681
7.18	0.34	2.28449	33.60	0.29	2.13264	1.18	0.01	0.25476
3.59	0.40	0.68404	16.80	0.11	3.39470	0.39	1 × 10^−5^	1.02124

^a b c^ The dose ratio of irinotecan and sunitinib in each cell line. The ratio of the drug combination was set according to the IC50 value of the single drug in each cell line.

## Data Availability

Not applicable.

## Supplementary Materials

The following are available online at https://www.mdpi.com/article/10.3390/cancers13143586/s1, Figure S1: The dose–effect relationships of seven single drugs on the growth of MCF-7, Figure S2: The dose–effect relationships of seven single drugs on the growth of MDA-MB-231, Figure S3: The dose–effect relationships of seven single drugs on the growth of SK-BR-3, Table S1: Details of the cancer data sets used in this work, Table S2: Gene dependency network related to the prognosis of BC, Table S3: Gene dependency network related to the prognosis of OV, Table S4: Gene dependency network related to the prognosis of GBM, Tables S5 and S6:The enriched pathways of essential genes in OV and GBM, Table S7: Drug combinations prediction for specific subtypes.
