# Supplementary material for: Systems Pharmacology-Based Precision Therapy and Drug Combination Discovery for Breast Cancer
Source: Cancers (Basel). 2021 Jul 17;13(14):3586. doi: 10.3390/cancers13143586 (PMC8305788; doi:10.3390/cancers13143586)
Supplement: Supplementary file 1 [file cancers-13-03586-s001.zip › cancers-1266866-supplementary proof.docx]

Supplementary Materials: Systems Pharmacology-Based
Precision Therapy and Drug Combination Discovery for Breast Cancer

Ze-Jia Cui, Min Gao, Yuan Quan, Bo-Min Lv, Xin-Yu Tong, Teng-Fei Dai, Xiong-Hui Zhou and Hong-Yu Zhang


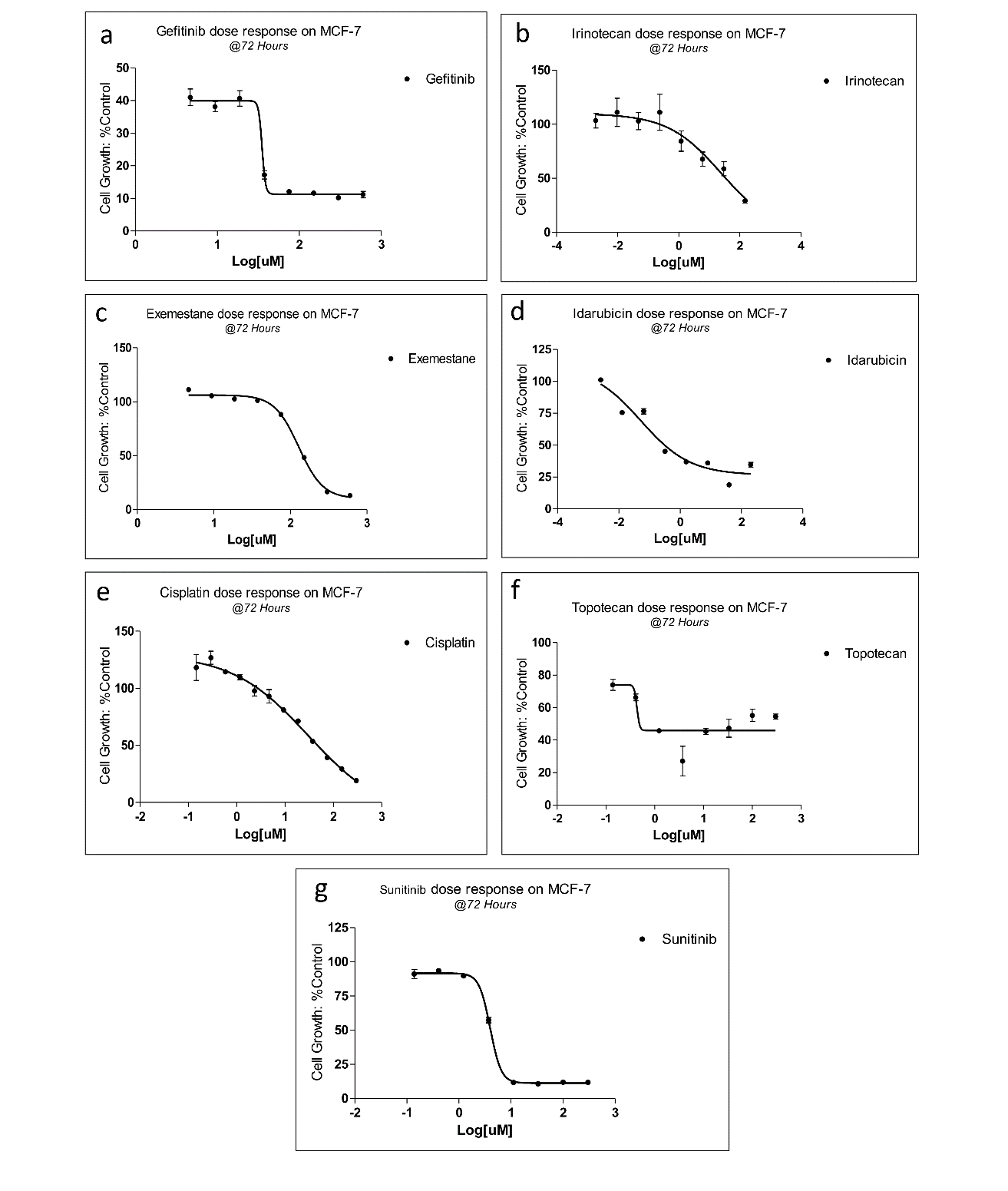


**Figure S1.**


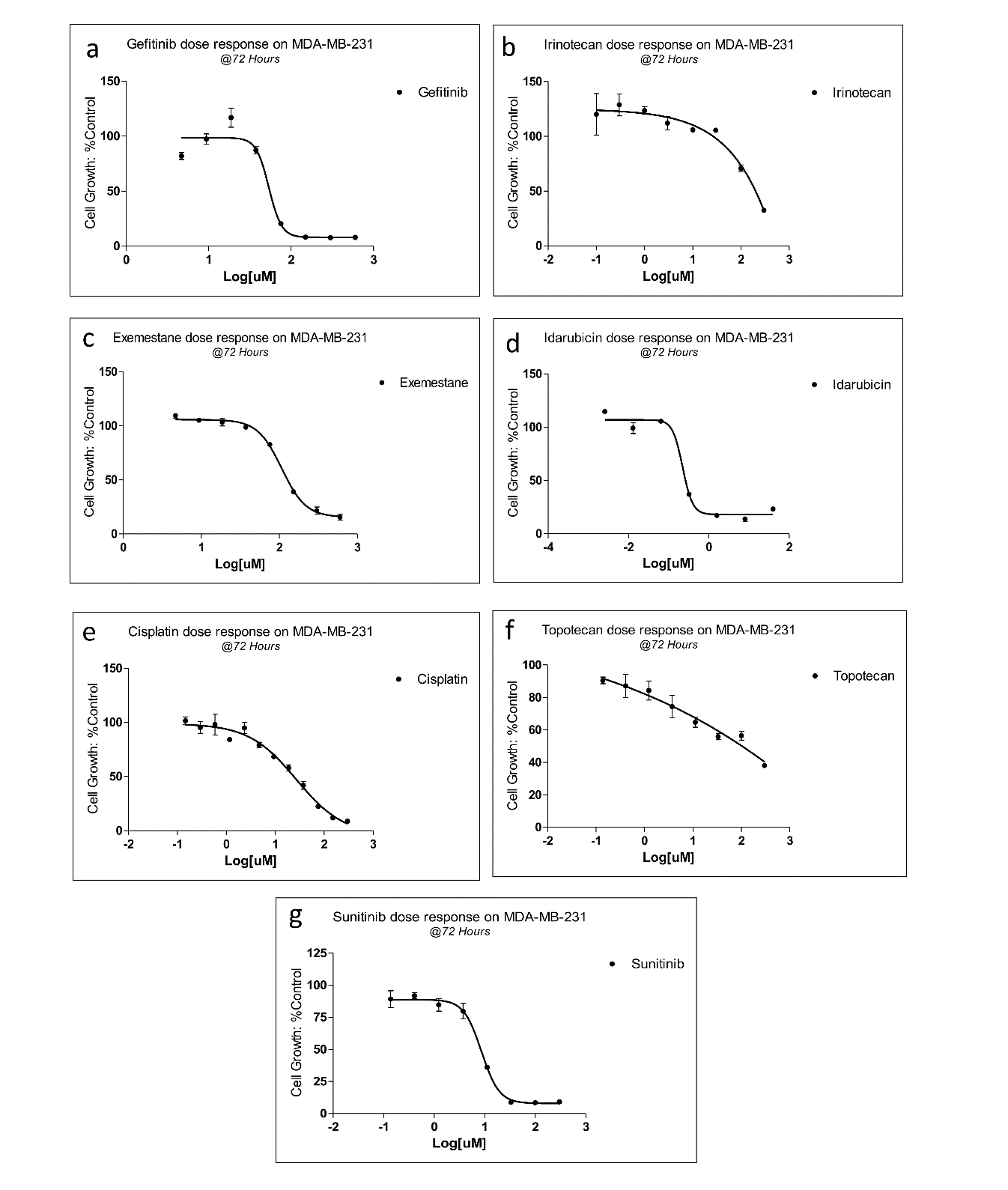


**Figure S2.**


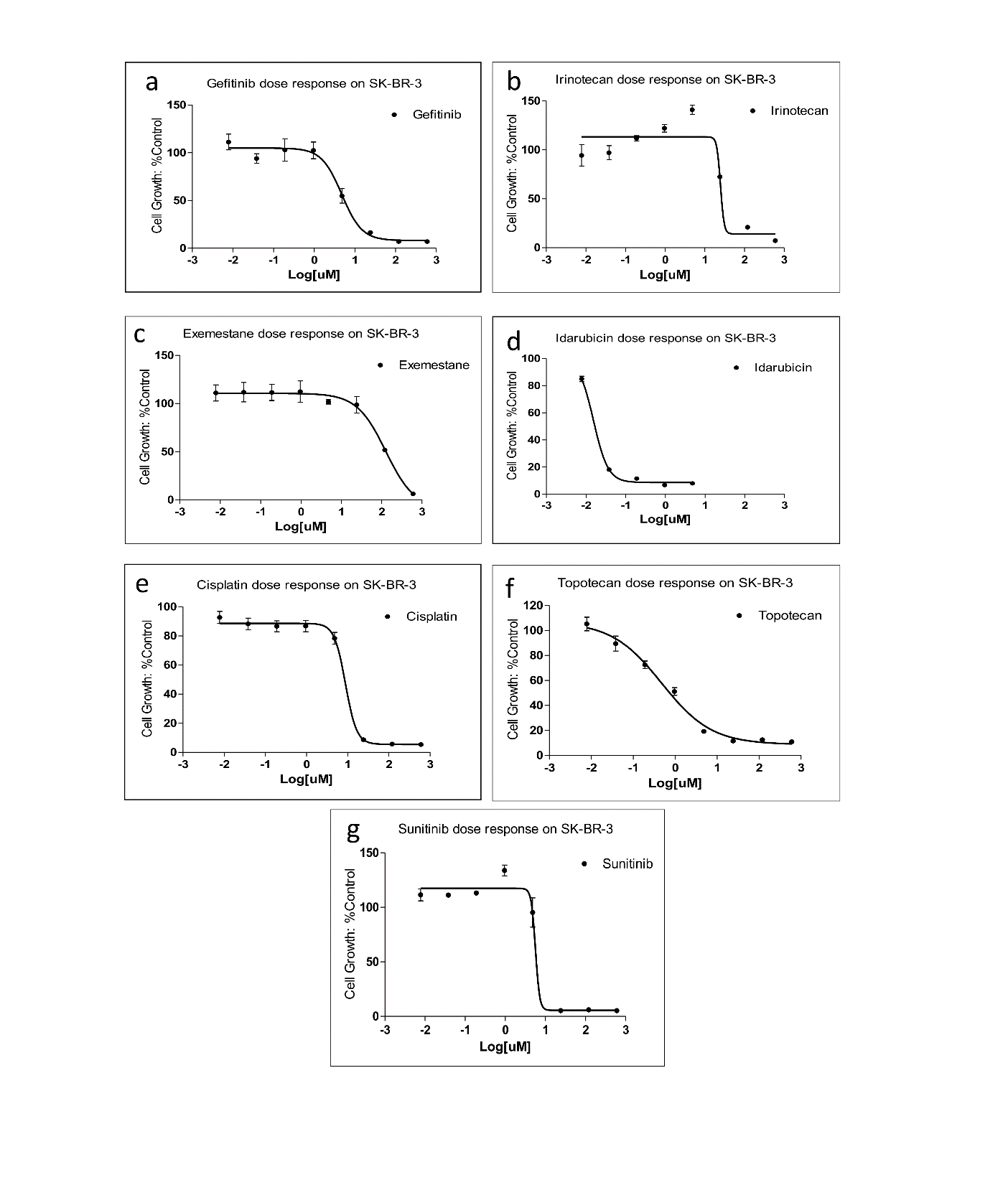


**Figure S3.**

**Table S1.** Details of the cancer data sets used in this work.

| **Data Sets** | **Platform** | **Number of Disease Samples** | **Number of Control Samples** | **Samples Used to Construct the Gene Dependency Network** | **Samples Used to Validate our Method** |
| --- | --- | --- | --- | --- | --- |
| BRCA | Illumina RNA-seq | 1109 | 113 | 356 | 647 |
| OV | Agilent gene-chips | 590 | 8 | 380 | 529 |
| GBM | Agilent gene-chips | 595 | 10 | 517 | 136 |

**Table S7.** The enriched pathways of essential genes in ovarian cancer.

| Gene Set Name | *p*-Value | FDR *q*-Value |
| --- | --- | --- |
| DNA replication | 3.21E-07 | 5.98E-05 |
| Neuroactive ligand-receptor interaction | 6.04E-06 | 3.48E-04 |
| T cell receptor signaling pathway | 7.38E-06 | 3.48E-04 |
| Olfactory transduction | 7.49E-06 | 3.48E-04 |
| Base excision repair | 4.80E-05 | 1.76E-03 |
| Renal cell carcinoma | 5.66E-05 | 1.76E-03 |
| B cell receptor signaling pathway | 9.31E-05 | 2.47E-03 |
| Focal adhesion | 1.44E-04 | 3.35E-03 |
| Nucleotide excision repair | 1.81E-04 | 3.39E-03 |
| Spliceosome | 1.82E-04 | 3.39E-03 |
| Pathways in cancer | 2.23E-04 | 3.78E-03 |
| Regulation of actin cytoskeleton | 2.91E-04 | 4.52E-03 |
| Butanoate metabolism | 4.40E-04 | 5.68E-03 |
| Leukocyte transendothelial migration | 4.53E-04 | 5.68E-03 |
| mTOR signaling pathway | 4.58E-04 | 5.68E-03 |
| Endocytosis | 8.41E-04 | 9.78E-03 |

**Table S8.** The enriched pathways of essential genes in glioblastoma multiforme.

| Gene Set Name | *p*-Value | FDR *q*-Value |
| --- | --- | --- |
| Regulation of actin cytoskeleton | 7.34E-09 | 1.15E-06 |
| Focal adhesion | 1.24E-08 | 1.15E-06 |
| Neurotrophin signaling pathway | 1.37E-07 | 8.14E-06 |
| Adherens junction | 1.75E-07 | 8.14E-06 |
| Chemokine signaling pathway | 8.28E-07 | 3.08E-05 |
| Wnt signaling pathway | 1.27E-06 | 3.94E-05 |
| Leukocyte transendothelial migration | 2.74E-06 | 7.29E-05 |
| Axon guidance | 6.96E-06 | 1.62E-04 |
| Dilated cardiomyopathy | 1.02E-05 | 2.11E-04 |
| Melanogenesis | 2.54E-05 | 4.73E-04 |
| Long-term potentiation | 5.44E-05 | 8.63E-04 |
| Cytokine-cytokine receptor interaction | 6.03E-05 | 8.63E-04 |
| MAPK signaling pathway | 6.03E-05 | 8.63E-04 |
| Peroxisome | 1.18E-04 | 1.57E-03 |
| Olfactory transduction | 1.95E-04 | 2.42E-03 |
| Glioma | 2.31E-04 | 2.68E-03 |
| Purine metabolism | 2.45E-04 | 2.68E-03 |
| Epithelial cell signaling in Helicobacter pylori infection | 3.07E-04 | 3.17E-03 |
| Oocyte meiosis | 3.37E-04 | 3.30E-03 |
| GnRH signaling pathway | 6.97E-04 | 6.48E-03 |
| Tight junction | 1.09E-03 | 9.61E-03 |
